# Critical events in intensive care unit

**DOI:** 10.4103/0972-5229.40947

**Published:** 2008

**Authors:** Mohandeep Kaur, Mridula Pawar, Jasvinder Kaur Kohli, Shailendra Mishra

**Affiliations:** **From:** Department of Anesthesia and Critical Care, Dr. RML Hospital, New Delhi

**Keywords:** Critical events, intensive care unit, outcome, reporting

## Abstract

This prospective study was designed to have an insight into critical events occurring in the 13-bedded multidisciplinary intensive care unit (ICU) of our hospital and to report the critical events to evaluate the avoidable/iatrogenic problems so as to improve patient outcome and keep a self-check in the ICU. The errors reported were due to wrong mechanical or human performance. Repeated performance errors of the same kind pointed to the problem area, to which was paid proper attention in the required manner. Some malfunctioning equipments were abandoned and the need for adequate availability of staff was emphasized. Reporting of critical events was done keeping the patients' and doctor's identities anonymous through a proforma designed to report the event.

## Introduction

An intensive care unit (ICU) is a continuously busy ward in which critically ill patients are on life support treatment under intensive monitoring. Doctors, nurses and technicians vigilantly work on the patients and handle the life support equipment, pipeline and monitors.

The concept of recording a critical event was adapted from studies in aviation psychology in the US Air-force during and after the World War II. It was extensively applied in anaesthesia by Cooper *et al*. in 1978.[1–2]

With this background, the present study was conducted to do an audit of reported critical events during intensive care stay and to develop a critical event reporting system in the ICU of our hospital.

Errors in the management of the patient may creep in due to various reasons. There is a very narrow zone to allow for any errors whatsoever in the ICU, as these may significantly increase the morbidity and mortality of critically ill patients.

Critical Event is any occurrence during the treatment of patient in an ICU, which if not detected and corrected in time would adversely affect the outcome of the patient. The importance of vigilant monitoring by a trained person is thereby emphasized.

## Materials and Methods

A prospective study of critical events was performed in a 13-bedded multidisciplinary ICU of Dr. RML Hospital, New Delhi over a period of 6 months, i.e., from January to June 2006. All the ICU beds are equipped with state-of-the-art monitors and ICU ventilators. Two beds have been earmarked as disaster beds and are kept vacant for serving the need of disaster as a policy of the hospital. The ICU is under supervision of the Department of Anaesthesia. The patients are admitted under medicine or surgery and are referred to ICU whenever an indication for ICU care arises. The protocol for ICU admission is fixed. Moribund/terminal patients are generally not brought to ICU as they are kept in wards on simple ventilators with basic modes. Patient-nurse ratio is fixed at three nurses for two patients, with an aim of keeping it as one for one. A consultant anaesthetist is incharge of ICU with a qualified resident anaesthetist on 12-h-shift duty. This study was designed to have a self-appraisal of ICU working. A proforma was designed for reporting critical events, keeping the patient and doctor identities anonymous. These proformas were collected in ICU in a special collection box. The box was emptied every 3 months and critical events were appraised. The ICU incharge had to encourage the resident doctors to report the events encountered during ICU duty. Critical event reporting was started as a practice when this study commenced and has been continued since then.

## Result

These events can be classified into mechanical errors and human errors. Mechanical errors could be due to ventilator, monitor or some other equipment failure. In an ICU, a patient remains continuously on life support for a prolonged period of time. During this period, equipments may sometimes malfunction due to various reasons. If this is not detected well in time, it may affect the patient outcome critically. In the present study, 29.62% events were due to mechanical errors and 70.37% due to human errors.

Observed critical events - *n* = 54

1. Mechanical errors i.e. ventilator failure - *n* = 16 (29.62%)
2. Human errors - *n* = 38 (70.37%)

**Table:** Proforma for critical events in intensive care unit

Age
Sex
Diagnosis
Status of the patient - whether on/off ventilator
On/off ionotropes
Any other relevant history/clinical detail
Critical event
Time
Details of event
Intervention done
Intervention done by whom
Observed by
Outcome observed
Expected outcome without intervention
Designation of reporter

Human errors included endotracheal tube-related problems and disconnection.

**Table:** 

**Human error**	***n* = 38**	**70.37%**
a) Extubation	16	29.62%
b) Intubation problem	4	7.40%
c) Blocked endotracheal tube	4	7.40%
d) Ventilator disconnection	6	11.11%
e) Oxygen disconnection from central pipe line	2	3.70%
f) Fail from bed	2	3.70%
g) Improper mode of ventilator	2	3.70%
h) CVP related	2	3.70%

Endotracheal tube-related errors were observed during:

- Extubation, which could either be self-extubation or inadvertent slipping out of the endotracheal tubes. This accounted for 29.62% of total human errors. The time at which these complications were observed could be related to bed-making or position-changing and back care in ICU. It shows that either the staff is not vigilant enough to take care of the endotracheal tube or it was not fastened well. In conscious patients, the hands should be kept tied so that self-extubation is not possible.
- Intubation, which could be either oesophageal or delayed due to difficult anatomy or untrained personnel attempting it. This accounted for 7.40% of total human errors.
- Endotracheal tube blockade, which could be because of thick secretions or kinking and it accounted for 7.40% of total human errors observed.

Disconnection-related errors were due to either ventilator disconnection (11.11% of total human errors) or disconnection of central oxygen supply to the ventilator (3.7% of total human errors). Both the causes of disconnection mentioned above accounted for 14.81% of total human errors.

Other human errors included fall from bed as the siderails were not raised after carrying out some the ICU procedure.

Faulty setting of ventilator and CVP-related problems were observed during the posting of new doctors in ICU.

Mechanical errors in our study included ventilator breakdown repeatedly with one make of ventilator. After every breakdown, the company people were informed. The problem would be rectified and the ventilator would work alright for a few days and would again break down. The ventilator undergoing repeated breakdowns was having a proximal flow sensor which would get stuck while ventilating the patient. Patients were put on standby portable ventilators whenever these breakdowns happened. Fortunately, no mortality was reported.

Medication errors often reported as adverse drug events (ADE) are another set of critical human errors in an ICU setting. Fortunately, in our ICU, no medication errors were observed which could be a matter of sheer chance.

## Discussion

Although critical events keep on happening in different ICUs, yet reporting of these events needs to be encouraged. Unless these events are reported, it would not be possible to develop a system to detect and overcome these. Reporting will also help to know the status and working profile of different ICUs and enable us to improve the existing status for better outcome of patients.

Harvard Medical Practice study II, published in 1991,[3] showed the nature of adverse events in hospitalized patients. It showed equipment and monitor malfunctions and human errors as the cause of adverse events. “Human errors” is by far the biggest risk and accounts for two-thirds of ICU complications. These complications contribute to hospital mortality.

A study conducted by Donchin *et al.*[4] brought to notice an estimated iatrogenic 1.7 error per patient per day (out of every 178 directed activities) in ICU. This study shows that human error is by far the biggest risk and accounts for two-thirds of ICU complications. Physicians had the highest rate of errors, though physicians and nurses were equal contributors to the total number of errors. Twenty-nine percent of these errors if uncorrected, had the potential to cause significant morbidity or even death.

Critical incident reporting in intensive care unit, published in 1997,[5] reported 281 critical incidents in a period of over 3 years. Detection of critical incidents in over 50% of cases resulted from direct observation of the patient, while monitoring systems accounted for another 27%. The most important events reported concerned airway management and invasive lines, tubes and drains. Human error was a factor in 55% of incidents, while violation of standard practice contributed to 28%.

The present study includes both equipment and ventilator malfunctions as human errors, as these should have been recognized by the ICU personnel. Non-recognition could be because of ignored alarms, inadequate knowledge about the alarms and their interpretation, which in itself implies inadequately trained staff or lesser vigilance due to understaffing in ICU. We encountered 70.3% human errors, which included 44.4% errors due to endotracheal tube-related problems like accidental extubation, which in the present study was mainly due to accidental slipping out of the endotracheal tube during bed-making at 8 a.m. or posture changes of the patient, which is scheduled every 3-hourly in our ICU. Blocked endotracheal tube and difficult intubation were the other endotracheal tube-related problems. The patients on ventilator are having disposable HME filters, which are changed everyday. Closed system suction catheter is used in all the intubated patients in our ICU. Every patient on ventilator is put on short-acting opioid (fentanyl) and benzodiazepine (midazolam) infusion or as bolus dose. The idea is to counter awareness in case of conscious patients who are intubated. Patients on elective ventilation who need to be weaned or assessed in the morning do not receive any sedation after 6 a.m. as per ICU protocol. Chest X-ray is done routinely in intubated patients. Senior resident on night duty manages the sedative requirements of these patients.

Intubation trolley is kept ready with all the essential requirements to handle difficult intubation. End tidal carbon dioxide is always available by the side of the patient. Senior resident on duty performs the intubation. ICU staff is fully trained to assist intubation.

In our study, the incidence of human errors due to ventilator events is almost 21% out of 70.3%; so this is a valid zone where improvement in alarm recognition and interpretation will be useful to prevent critical events in ICU. That is why new doctors and nurses posted in ICU are given an observer posting for 15 days prior to independent posting to make them aware of ICU working, equipments and protocols.

Mechanical ventilators generate alarms for patient disconnection or for some critical ventilator events due to ICU procedures; circuit obstruction by condensed water or undetermined factors or extubation. In the study of Evans *et al.*,[6] they designed new audio/video ventilator alerts distinct from other alarms in ICU which were impossible to ignore. Patient safety was increased by these enhanced alerts, which alerted all medical staff in ICU of all critical ventilator events in a timely manner.

In case of repeated ventilator failures observed in one specific type of ventilator in our ICU, a decision was taken to stop using those ventilators and written information was sent to the purchase department to abandon future purchase of this ventilator in our hospital.

A study sponsored by HHS agency for healthcare research and quality ‘The Critical Care Safety Study’, published in Critical Care Medicine Aug 2005, mentioned that 20% of patients admitted to Medical ICU and Coronary Care Unit experienced adverse events as these patients are among the sickest, they may be more vulnerable to errors in care and therefore more susceptible to injury of these events. Over 90% of all incidents occurred during routine care, out of which 45% were preventable.

A study of preventable adverse drug events in hospitalized patients comparing intensive care and general care units, published in 1997,[7] mentions that ICU patients receive up to 50% more drugs than their general medical or surgical cohorts; so they have a greater likelihood of experiencing an adverse drug reaction (ADR). Preventable adverse drug events and potential adverse drug events occur at a rate of 19 per 1000 patient ICU days, a rate twice that of non-ICU-care wards.

In another study on systems analysis of adverse drug events published in 1995,[8] 75% of drug errors are preventable, out of which 39% were due to wrong ordering by the physician and 38% were due to wrong administration by the nurse.

However, fortunately in our study, we did not observe any adverse drug event in the ICU, which could be a matter of sheer chance. However, it is one of the major critical events in an ICU.

## Inferences of the Study

1. Junior doctors and new nurses joining the ICU need to be trained in the equipment usage and management protocols of ICU. They should handle the patients and equipment under the guidance of trained people in the beginning. This is important to safeguard against the failed intubations or accidental extubations. So, new personnel should first be posted as observer for 1-2 weeks in the ICU.
2. New senior residents were encouraged to report and document the critical events to ICU incharge immediately, as the prompt action taken may sometimes reverse the adverse effects of critical events. So, ICU work should be considered a team work and nothing should be hidden from each other. Basically, the decision taken to abandon the use of ventilators of one particular type was the result of this documentation and analysis.
3. Most of the accidental extubations occur during bedding and repositioning of the patient during nursing care. So, care should be taken to avoid extubation, disconnection and malpositioning of the tube. A senior nurse was therefore told to supervise bedding and posture-changing of the patient.
